# Long-term clinical efficacy and safety of thalidomide in patients with transfusion-dependent β-thalassemia: results from Thal-Thalido study

**DOI:** 10.1038/s41598-023-40849-4

**Published:** 2023-08-21

**Authors:** Zahid Ali, Mohammad Ismail, Inayat Ur Rehman, Gulab Fatima Rani, Muhammad Ali, Muhammad Tariq Masood Khan

**Affiliations:** 1 Department of Pharmacy, University of Peshawar, Peshawar, Khyber Pakhtunkhwa Pakistan; 2 Department of Pharmacology, Northwest School of Medicine, Peshawar, Khyber Pakhtunkhwa Pakistan; 3 Department of Pathology, Institute of Pathology and Diagnostic Medicine, Khyber Medical University Peshawar, Peshawar, Pakistan; 4 Department of Hematology, Pak International Medical College, Peshawar, Khyber Pakhtunkhwa Pakistan; 5 Blood Disease Clinic, Peshawar, Khyber Pakhtunkhwa Pakistan

**Keywords:** Diseases, Haematological diseases, Anaemia

## Abstract

Regular blood transfusion is the mainstay of treatment in transfusion-dependent β-thalassemia (TDT); however, transfusions culminate in an array of serious complications. Therefore, a single-arm, non-randomized clinical trial was conducted in hydroxyurea refractory TDT patients to explore the long-term safety and efficacy of thalidomide. The primary outcomes for efficacy were rise in hemoglobin (Hb) level and changes in transfusion frequency. Whereas, several clinical and laboratory parameters were assessed for safety of thalidomide. Secondary outcomes included changes in serum ferritin, serum lactate dehydrogenase (LDH), serum uric acid, red blood cell indices, and size of liver and spleen. A total of 532 patients were followed for a period of 30 months. Significant increase in mean Hb level was identified at 6 months (1.4 g/dL, p ≤ 0.001) and 30 months (2 g/dL, p ≤ 0.001) in comparison with baseline. A total of 408 (76.7%) patients responded to thalidomide therapy (excellent responders 25.8%, good responders 31%, and partial responders 19.9%) and attained transfusion independence within 6 months of therapy. A significant decline in mean ferritin, LDH level, liver size, and spleen size was observed. No unfavorable effects were observed on kidney and liver functions. Mild adverse events were reported in 48 (9%) patients and serious adverse events, including cerebral vascular accident and portal vein thrombosis were reported in two patients each. This study concludes that thalidomide is an effective and well-tolerated drug that can improve Hb levels and reduce transfusion burden in hydroxyurea refractory TDT patients.

**Trial registration:** This trial is registered at http://www.clinicaltrial.gov as # NCT03651102.

## Introduction

Thalassemia is a common single gene hereditary disease, characterized by insufficient hemoglobin (Hb). Based on the globin gene involved, thalassemia may generally be classified as α-thalassemia or β-thalassemia^1^. In β-thalassemia, Hb production is decreased because of mutations or deletion of the HBB gene, causing an imbalance in the globin chains (underproduction of β-globin chains and excess of α-globin chains). The excess free α-globin chains precipitate in the cell resulting in premature cell death and consequently ineffective erythropoiesis^2^.

β-thalassemia, on the basis of blood transfusion requirement, is classified into transfusion-dependent β-thalassemia (TDT) and non-transfusion-dependent β-thalassemia (NTDT). Lifelong and regular blood transfusions are required for TDT patients, while NTDT patients require only occasional blood transfusions, if any^2,3^.

At present, β-thalassemia seriously threatens public health, and the main therapies for patients with β-thalassemia include regular blood transfusion and iron chelation. However, chronic blood transfusion poses a significant risk of iron overload and subsequent multi-organ damage, as well as an increased risk of transfusion-related infectious diseases and acute life-threatening events such as acute hemolytic reactions, and anaphylaxis^4^. Hematopoietic stem cell transplantation (HSCT) is considered the primary curative therapeutic modality; however, lack of access to bone marrow transplant services, high degree of human leukocyte antigen (HLA) polymorphism, availability of donors with perfectly matched HLA-antigens and the exorbitant cost delimits its applicability. Gene therapy and gene editing are other such options with rather insubstantial feasibility, especially in developing countries^3^.

Owing to the risks and limitations of current treatment options, fetal hemoglobin (HbF) inducers have markedly attracted clinicians’ attention in recent years. A number of studies have demonstrated the widespread use of hydroxyurea (HU) in patients with β-thalassemia because of its HbF inducing properties^3,5^. HU appears to be a safe, effective, and well tolerated drug in thalassemia^6^. A significant number of patients, however, either do not respond to HU therapy upfront or develop refractoriness over time^7^. Therefore, patients who do not respond to HU have the highest medical needs and are probably a group that deserves the highest priority; new interventions are eagerly required in this group of patients.

Other therapeutic options in TDT and NTDT patients include DNA methyl-transferase inhibitors and histone deacetylase inhibitors which can also induce HbF production, however, their safety and efficacy is yet to be determined^8,9^.

Recently, drug repositioning, also referred to as drug repurposing or re-tasking has gained recognition as an alternative strategy to discover new pharmacological effects of an existing drug that has well-proven pharmacokinetics and safety in humans^10,11^. Drug repositioning results in lower drug development costs, timeline, and risk of failure of a clinical trial due to adverse events. Furthermore, this approach has helped establish novel use for several drugs such as sildenafil, once used as anti-hypertensive, is now used to treat erectile dysfunction, and aspirin for prevention of myocardial infarction.

Thalidomide, a well-known drug, has previously been repositioned two times with success (first in 1998, for complications of leprosy and then in 2006, for multiple myeloma as first line drug)^10^. The drug has demonstrated promising response in thalassemia in terms of alleviating transfusion requirement in TDT patients by inducing HbF^3^. However, long-term safety remains a primary concern. A number of studies have reported a significant improvement in Hb levels among TDT patients with thalidomide therapy^3,12–22^. However, long term safety and efficacy in a large group of TDT patients, is yet to be determined. The current study, thus, aimed at elucidating this gap among HU refractory TDT patients.

## Methods

### Study design and setting

This single arm, non-randomized, off-label clinical trial was approved by the Ethics Committees at University of Peshawar (Ref No. 507/EC-FLES-UOP/2022) and Northwest General Hospital & Research Centre Peshawar (Ref No. NWGH/DMER/EC/07-1) and is in compliance with the ethical princliples of Declaration of Helsinki. The study was conducted at Blood Diseases Clinic (BDC) Peshawar from January 2018 till December 2020. This trial is registered at http://www.clinicaltrial.gov as # NCT03651102 dated 29/08/2018.

### Inclusion and exclusion criteria

The study included diagnosed HU refractory TDT patients of either gender, aged ≥ 18 months. Patient/parent/guardian was educated regarding the course of thalidomide therapy, and then full informed consent was obtained. Patients who remained transfusion-dependent despite using HU for ≥ 6 months were considered HU refractory. TDT diagnosis was ascertained by obtaining a valid Hb electrophoresis and/or HPLC report performed pre-transfusion at an age of 6 months or older, suggestive of β-thalassemia syndrome. Alternatively genetic testing profile (comprising PCR or HBB gene sequencing) suggestive of β-thalassemia syndrome was also regarded a diagnostic strategy. Patients with NTDT, viable pregnancy or planning conception, prescribed erythropoiesis stimulating agents for ≤ 3 months before enrollment, hemoglobinopathies other than β-thalassemia, altered liver and kidney function, thrombotic predisposition, history of neurological problems, cardiac disease, pulmonary deficits, cytopenias and/or active infection were deferred/excluded from the study. Enrolled female patients were well informed that they should absolutely avoid pregnancy during the study period and until 6 months after thalidomide withdrawal.

### Thalidomide intervention

All patients were given oral thalidomide, starting with a dose of 1–2 mg/kg/day. Among those with sub-optimal or no response, the dose was escalated in increments of 1 mg/kg/day at 2 months interval to a maximum of 4 mg/kg/day (maximum dose 70 mg/day). The drug was discontinued once it was administered at ≥ 2 mg/kg/day for a period of 6 months with no response. Patients were also continued on HU at a dose of 10-20 mg/kg/day. Additionally, clopidogrel at a dose of 2–4 mg/kg/day was administered to prevent the risk of thrombosis. Patients were also continued on iron chelation therapy in case of iron overload. Moreover, patients received dietary supplements, anti-histamines and hepatoprotective agents as supportive care. Blood transfusions were administered to patients who exhibited hemodynamic instability or were unable to maintain Hb level at 6 g/dl or above.

### Treatment response criteria

Definitive response to thalidomide intervention was evaluated at 6th month of therapy employing the following criteria: Excellent Response (ExR): maintenance of Hb level ≥ 9 g/dL without blood transfusion in last ≥ 56 days, good response (GR): maintenance of Hb level in the range of 7–8.9 g/dL without blood transfusion in last ≥ 56 days, and partial response (PR): maintenance of Hb level in the range of 6–6.9 g/dL without blood transfusion in last ≥ 56 days. Whereas, no response (NR): maintenance of Hb level < 6 g/dL. Blood transfusion was administered, if at any stage Hb level dropped below 6 g/dL. Among the non-responders, thalidomide was discontinued after 6 months of treatment, and were continued on blood transfusion therapy. In this study, Hb level of 6 g/dL as the cutoff was based on the understanding that a robust erythropoietic drive is crucial for achieving the best possible response, for which anemia serves as a natural stimulus. Besides, studies also suggests 6 g/dL as the minimum tolerable Hb level in thalassemia syndromes^23,24^. However, patients were strictly monitored for hemodynamic instabilities such as tachycardia, dyspnea, chest discomfort, frequent palpitations, syncope or fainting, lower limb oedema, fatigue or exercise intolerance, growth retardation and visceromegaly. Patients with any of these symptoms were started on regular transfusions.

### Primary treatment outcomes

I. Efficacy: Determination of rise in Hb level and transfusion independence.
II. Safety: Assessment of liver function test, kidney function test and adverse events particularly thromboembolic event or neuropathy.

### Secondary treatment outcomes

I. Determination of change in RBC indices, serum ferritin, LDH and uric acid levels.
II. Determination of change in liver and spleen size after thalidomide treatment.

Demographic details, clinical history and a thorough clinical examination was tackled by an experienced hematologist. Venous blood sample (6 mL) was collected under aseptic conditions in EDTA containing and gel sample collection tubes. The findings were recorded on a structured data collection form at baseline and on monthly basis for a period of 30 months. Blood samples were collected in two different sample collection tubes: Plastic Serum Tube with silicone coated interior (red top) 4 mL (catalog # 367812; BD Vacutainer^®^. Plastic Whole Blood Tube with spray-coated K_2_EDTA (lavender top) 2 mL (catalog # 367841; BD Vacutainer^®^). The collected samples were then tested for complete blood count, serum ferritin, serum LDH, serum creatinine, serum bilirubin, serum alanine transaminase (ALT), and serum uric acid to assess the primary and secondary outcomes of the study.

To assess the efficacy of thalidomide therapy, hematological profile (complete blood count), radiological findings (hepatomegaly and splenomegaly), and serum biochemical profile (uric acid, LDH, and ferritin levels) were assessed at baseline and on monthly basis throughout the study duration.

For safety of thalidomide therapy, liver function tests (bilirubin and ALT) and renal function tests (creatinine) were assessed at baseline and on monthly basis throughout the study duration. Additionally, adverse events were recorded on each follow-up to determine the safety of thalidomide therapy. The patient/guardian/parent was specifically inquired regarding paresthesia, rash, constipation, unwarranted infections, bleeding symptoms, pain in the abdomen, headache, syncope, focal weakness, and behavioral changes.

### Statistical analyses

All statistical analyses were performed using SPSS 23.0 (Chicago, IL, USA) and p-values < 0.05 were considered statistically significant. Patients were classified into responders and non-responder based on Hb level and transfusion requirement. Continuous data was presented as means ± SD and categorical data was presented as frequencies and percentages. A paired sample t-test was used to compare changes in continuous variables such as Hb, RBC, and its indices, serum ferritin, serum LDH, and uric acid levels, liver and spleen size, bilirubin, ALT and creatinine levels before and after thalidomide treatment.

## Results

### Patient flow chart

**Figure 1:**
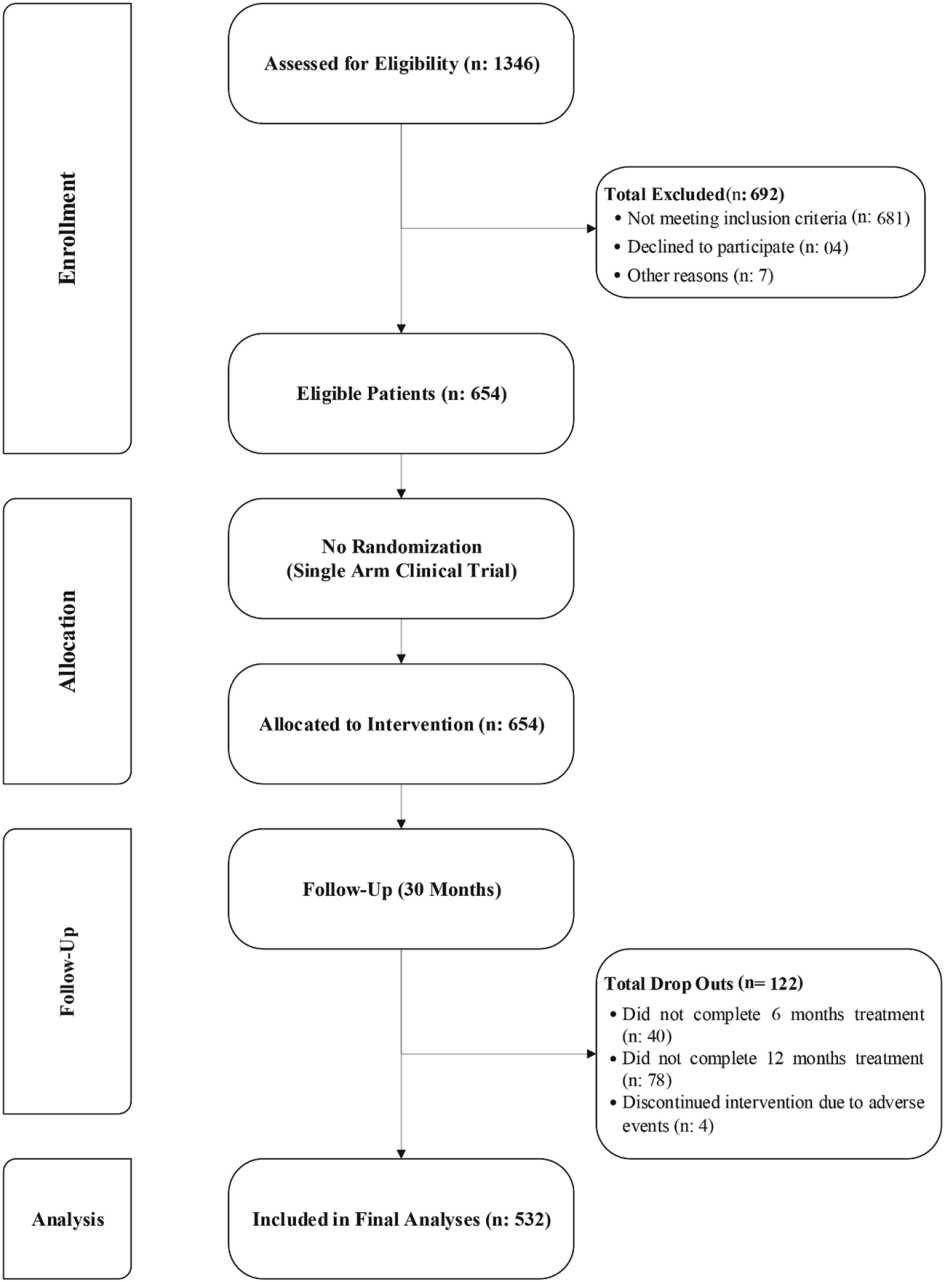
Schematic flowchart of patient inclusion.

A total of 1346 patients were screened for eligibility, of which 654 patients were eligible and included in the study. Drop out during the study period was 122 patients, of which 40 patients did not complete 6 months of thalidomide treatment, 78 patients were lost to follow-up after 6 months and did not complete 30 months of treatment and four patients discontinued treatment due to adverse events. Thus, a total of 532 patients were included in the final analyses (Fig. 1).

### General characteristics

The general characteristics of the included patients are presented in Table 1. Of the 532 patients included in the final analyses, 353 (66.4%) were male. The mean age of TDT patients was 5.3 ± 3.8 years (range = 1.5–26 years) with a mean weight of 15.1 ± 7.2 kg. Of the included patients, 5 (0.9%) patients were adult and 82 (15.4%) were splenectomized. The mean age at first transfusion of the included patients was 11 ± 22.8 months, while, the mean transfusion interval at baseline was 26 ± 25 days. However; a subset of patients, accounting for 16.7% of the total patients, exhibited a notably short transfusion frequency (1–10 days) which was attributed to hypersplenism at baseline. Family history of thalassemia was positive among half of the patients (50.4%).

**Table 1:** General characteristics.

Parameter	Category	n (%)	Means ± SD
Gender	Male	353 (66.4)	–
	Female	179 (33.6)	
Age (years)	≤ 2	129 (24.2)	5.3 ± 3.8
	3–4	115 (21.6)	
	5–6	97 (18.2)	
	7–8	100 (18.8)	
	> 8	91 (17.1)	
Weight (kg)	≤ 10	165 (31)	15.1 ± 7.2
	11–15	163 (30.6)	
	16–20	110 (20.7)	
	> 20	94 (7.7)	
Location	Afghanistan	63 (11.8)	–
	Khyber Pakhtunkhwa	453 (85.2)	
	Punjab	11 (2.1)	
	Baluchistan	4 (0.8)	
	Azad Jammu Kashmir	1 (0.2)	
Family history	Positive	268(50.4)	–
	Negative	264 (49.6)	
Age at first transfusion (months)	1–5	184 (34.6)	11 ± 22.8
	6–10	180 (33.8)	
	11–15	85 (16)	
	> 15	83 (15.6)	
Transfusion frequency (days)	1–10	89 (16.7)	25.7 ± 25
	11–20	120 (22.6)	
	21–30	194 (36.5)	
	> 30	129 (24.2)	
Splenectomy	Yes	82 (15.4%)	–
	No	450 (84.6%)	

### Treatment outcomes

**Figure 2:**
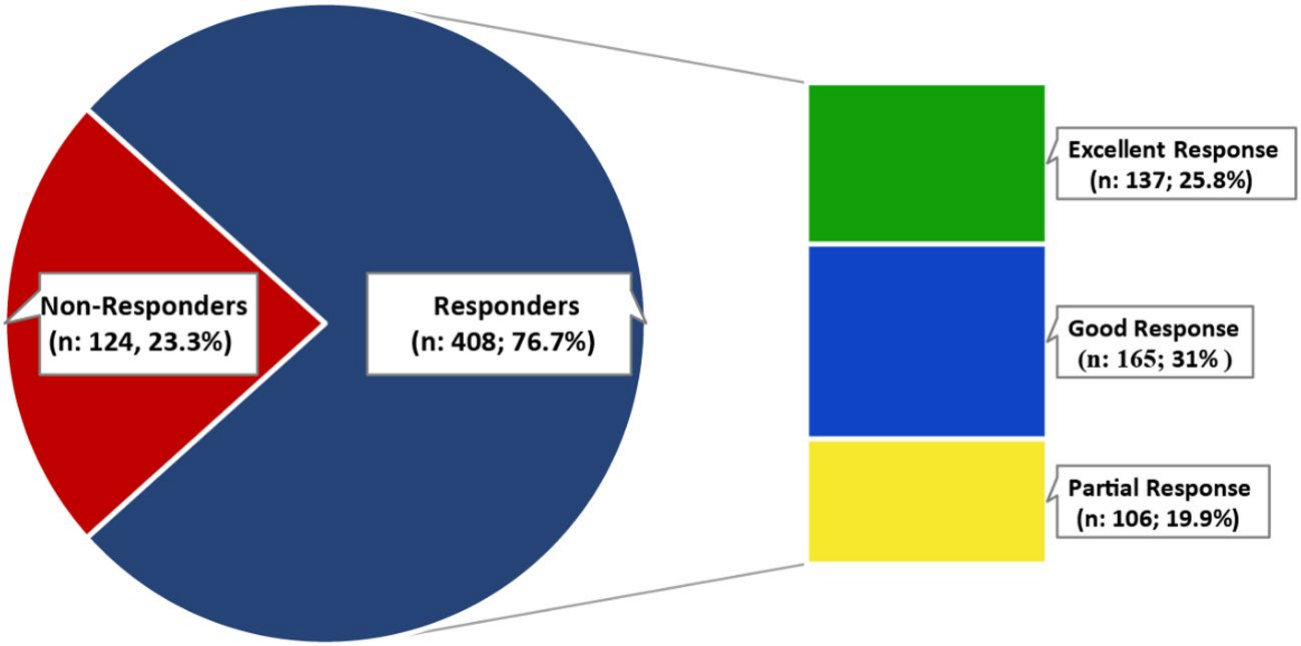
Hematological response of TDT patients upon thalidomide therapy.

The response to thalidomide therapy at 6th month of treatment revealed that of total of 532 patients, 408 (76.7%) patients exhibited positive responses and achieved transfusion independence (Fig. 2). Whereas, 124 patients (23.3%) were non-responsive to thalidomide therapy and remained transfusion-dependent. Among responders, 137 (25.8%) patients demonstrated excellent response, 165 (31%) exhibited good response and 106 (19.9%) patients were partial responders. Regarding the thalidomide dosage in different response groups, the mean dose was 1.9 mg/kg/day in excellent responders followed by 2.1 mg/kg/day in good responders, and 2.6 mg/kg/day among partial responders. Whereas, non-responders were taking thalidomide in a mean dose of 2.8 mg/kg/day.

#### Impact of thalidomide therapy on hemoglobin and red blood cell indices

**Figure 3:**
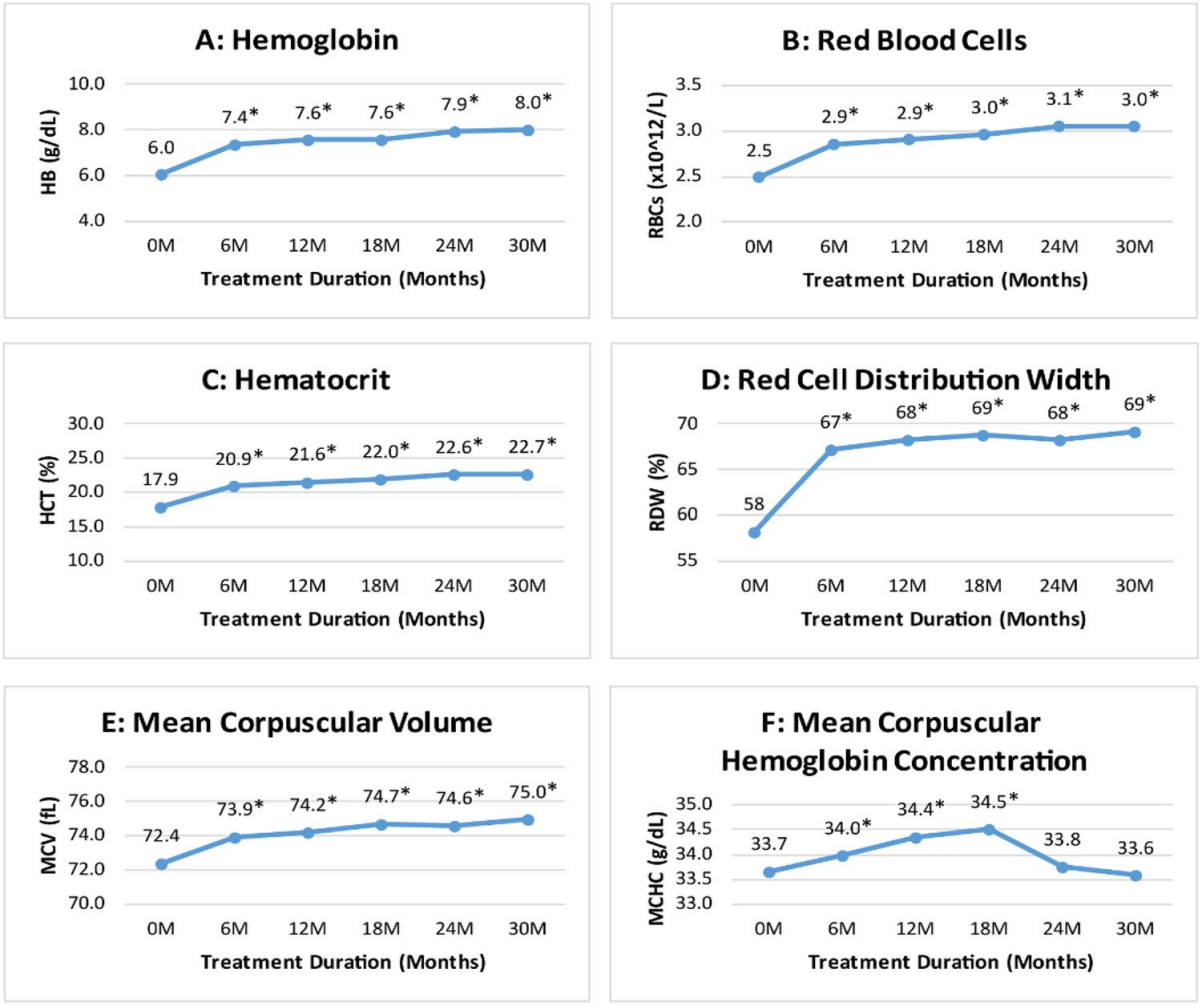
Change in hemoglobin and RBC indices at different time intervals. *Indicates significance at p-value of < 0.05.

The effect of thalidomide on Hb level over 30 months of therapy showed that the Hb level significantly increased from a mean level of 6 g/dL at baseline to 7.4 g/dL at 6th month (p ≤ 0.001) and 8 g/dL at 30th month (p ≤ 0.001) of the treatment (Fig. 3). Likewise, the RBC count, p ≤ 0.001), hematocrit (p ≤ 0.001), mean corpuscular volume (MCV, p ≤ 0.001), and red cell distribution width (RDW, p ≤ 0.001) improved significantly at 6th and 30th month of thalidomide therapy compared to baseline. Mean corpuscular hemoglobin concentration (MCHC) compared to baseline (33.7 g/dL) significantly increased at 6th month (34 g/dL, p ≤ 0.001) till 18th month of treatment (34.5 g/dL, p ≤ 0.001), however, no significant increase was observed at 30th month of thalidomide treatment (p = 0.481).

#### Impact of thalidomide therapy on white blood cell and platelets

**Figure 4:**
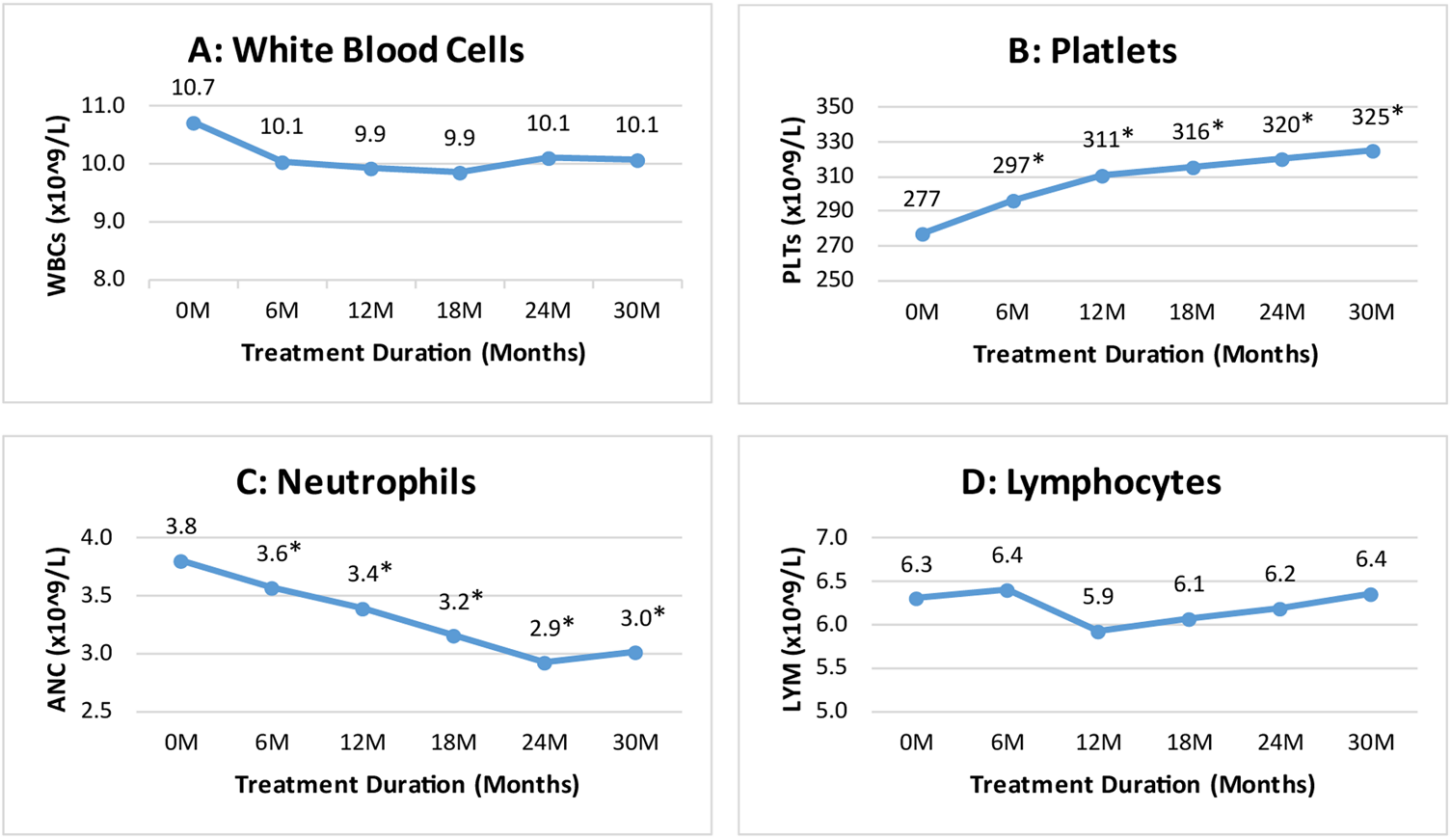
Change in WBC and platelets levels at different time intervals. *Indicates significance at p-value of < 0.05.

Our study showed that the absolute neutrophil count (ANC) significantly reduced from a mean level of 3.8 ± 0.8 × 10^9^/L at baseline to 3.6 ± 1.1 × 10^9^/L at 6th month (p ≤ 0.001) and 3.0 ± 1.2 × 10^9^/L at 30th month (p ≤ 0.001) of thalidomide therapy (Fig. 4). The platelet count increased significantly from 277 ± 136.7 × 10^9^/L at baseline to 297 ± 149.7 × 10^9^/L at 6th month (p = 0.028) and 325 ± 208.2 × 10^9^/L at 30th month (p ≤ 0.001) of therapy. However, no significant changes were observed in total leukocyte count (p = 0.209) and lymphocytes (p = 0.877) throughout the study duration.

#### Impact of thalidomide therapy on serum ferritin, LDH, liver and spleen size

**Figure 5:**
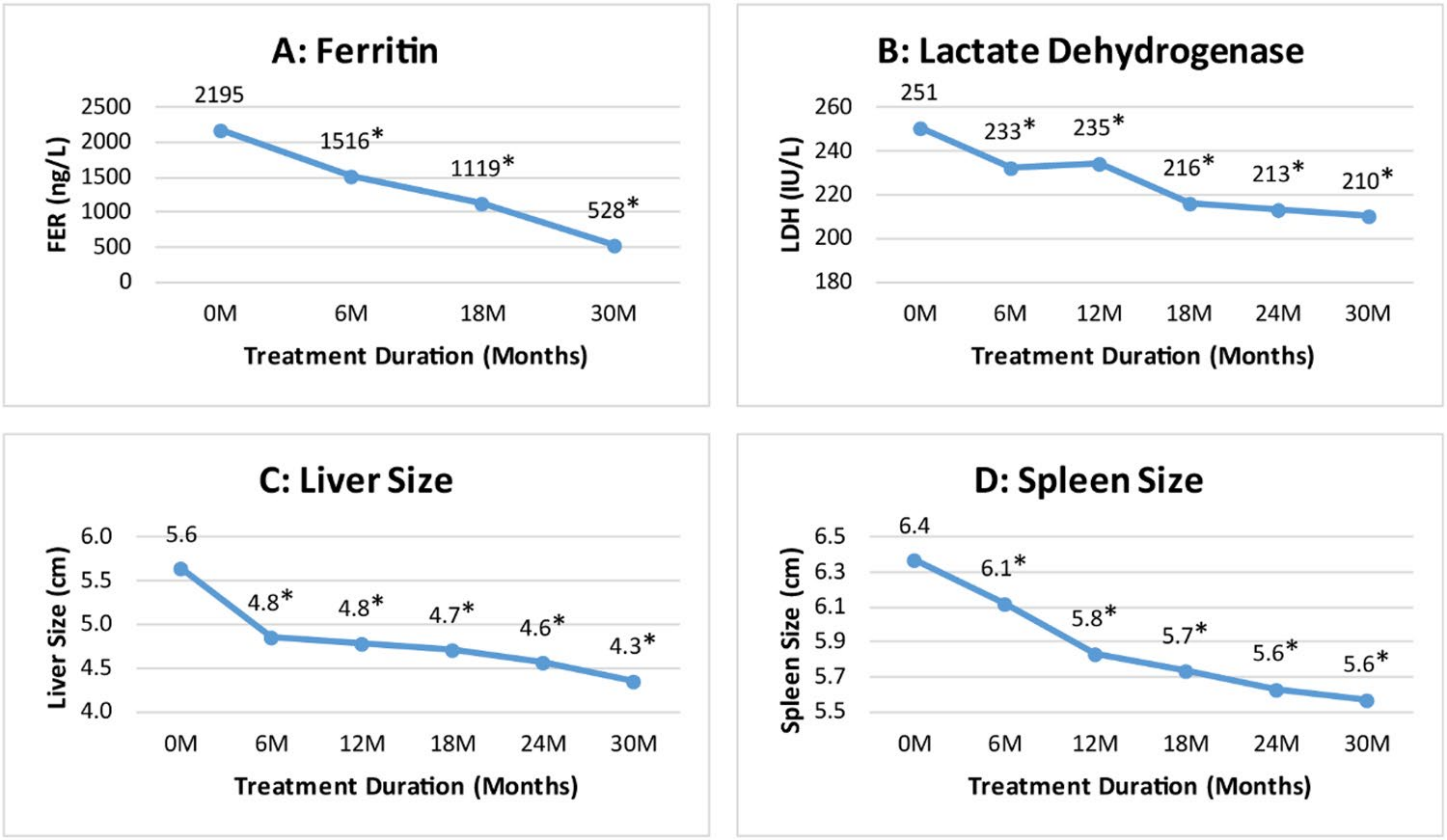
Change in serum ferritin, LDH, spleen and liver size at different time intervals. *Indicates significance at p-value of < 0.05.

Our data revealed a significant decline in serum ferritin levels from a mean of 2195 ng/mL µg/L at baseline to 1516 ng/mL at 6th month (p ≤ 0.001), and 528 ng/mL at 30th month (p ≤ 0.001) of thalidomide treatment (Fig. 5A). Likewiese, LDH level significantly decreased from a mean of 251 ± 12 IU/L at baseline to 233 ± 25 IU/L at 6th month (p = 0.001), and 210 ± 7 IU/L at 30th month of thalidomide therapy (p ≤ 0.001) (Fig. 5B). The liver size reduced significantly from 5.6 ± 2.6 cm at baseline to 4.8 ± 2.1 cm at 6th month (p ≤ 0.001), and 4.3 ± 1.8 cm at 30th month (p ≤ 0.001) of thalidomide therapy (Fig. 5C). A similar pattern was observed for spleen size, which significantly reduced from 6.4 ± 3.3 cm at baseline to 6.1 ± 3.3 cm at 6th month (p ≤ 0.001), and 5.6 ± 3 cm at 30th month (p ≤ 0.001) of thalidomide therapy (Fig. 5D).

#### Impact of thalidomide therapy on liver and renal function

**Figure 6:**
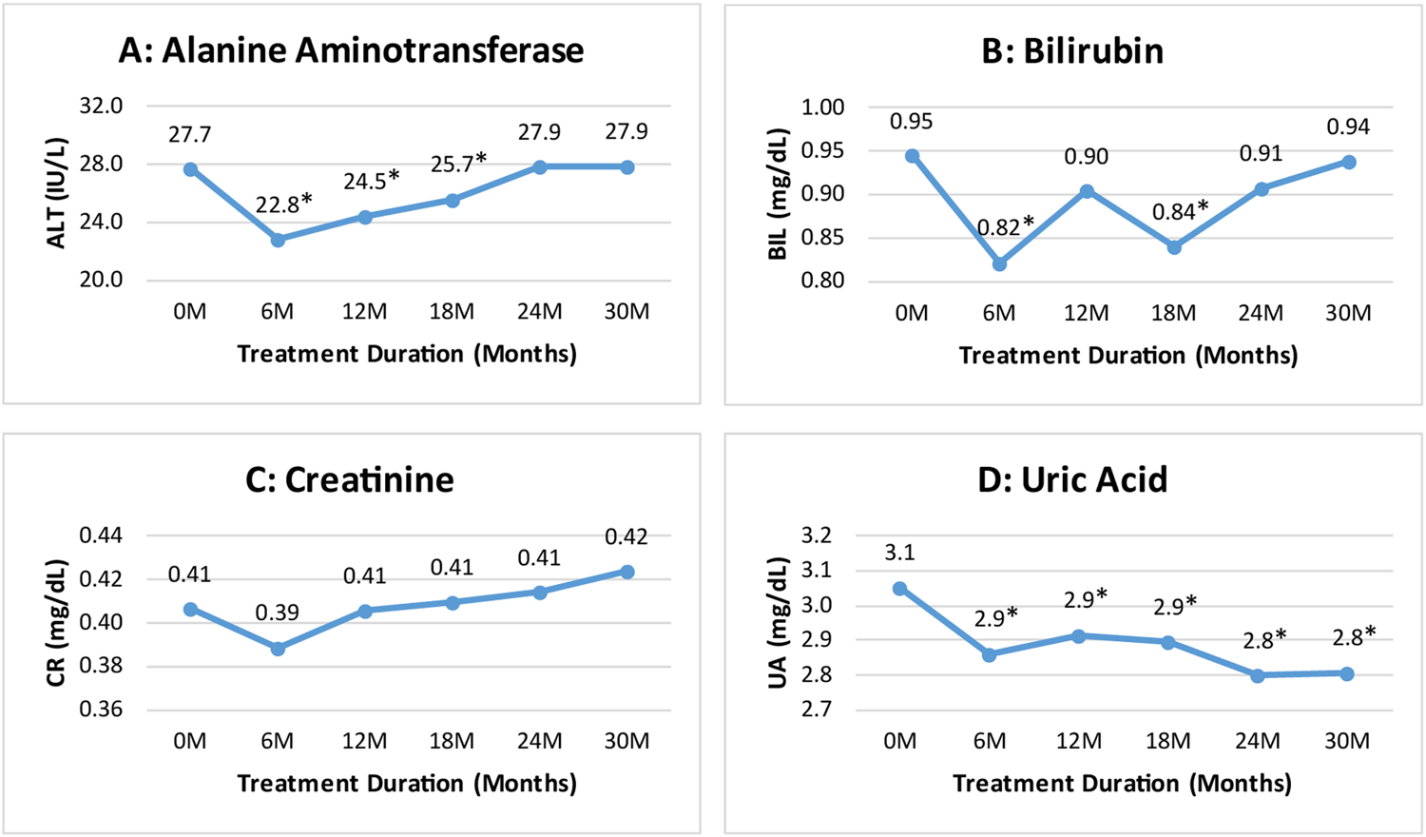
Changes in ALT, bilirubin, creatinine and uric acid levels at different time intervals. *Indicates significance at p-value of < 0.05.

The serum biochemical findings concerning the hepatic and renal safety of thalidomide therapy revealed that the mean level of ALT reduced significantly (p ≤ 0.001) from 27.7 ± 20 IU/L at baseline to 22.8 ± 5.8 IU/L at 6th month of therapy, but no significant changes in the mean level of ALT were observed at 30th month of therapy when compared with baseline (Fig. 6A). Similarly, the mean bilirubin level reduced significantly from 0.95 ± 0.3 mg/dL at baseline to 0.82 ± 0.3 mg/dL at 6th month of therapy (p ≤ 0.001), but no significant change was observed in the mean bilirubin level at 30th month of therapy when compared with baseline (Fig. 6B).

Our data revealed no significant changes in the mean creatinine levels throughout the study duration (Fig. 6C). Whereas, a significant decline in the mean uric acid level was observed from 3.1 ± 0.6 mg/dL at baseline to 2.9 ± 0.4 mg/dL at 6th month (p ≤ 0.001) and 2.8 ± 0.4 mg/dL at 30th month (p ≤ 0.001) of thalidomide therapy as compared with baseline (Fig. 6D).

#### Thalidomide-related adverse events

**Figure 7:**
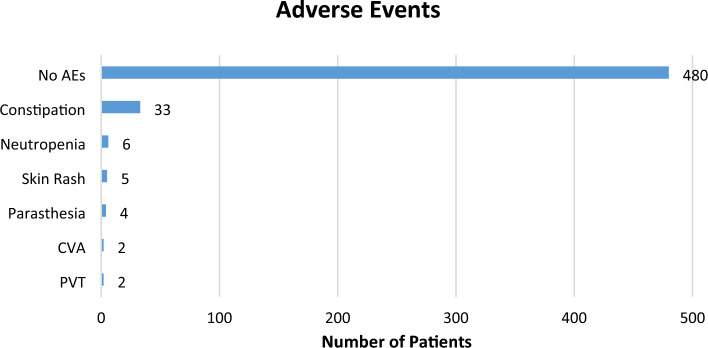
Incidence of adverse events in TDT patients during thalidomide therapy***.*** *ADRs* adverse drug reactions, *CVA* cerebral venous accident, *PVT* portal vein thrombosis.

Overall, the incidence of adverse events was reported in n = 52 (9.8%) patients over the course of 30 months of thalidomide therapy (Fig. 7). The most frequent adverse event was constipation (n = 33, 6.2%), neutropenia (n = 6, 1.1%), skin rash (n = 5, 0.9%), and paresthesia (n = 4, 0.8%). Thalidomide was discontinued in four patients following cerebrovascular accident (CVA, n = 2) and portal vein thrombosis (PVT, n = 2).

## Discussion

The present study evaluated the long-term safety and efficacy of thalidomide therapy with encouraging results in a relatively large number of TDT patients. Thalidomide, compared to transfusions, is more convenient and economical because transfusions induce iron overload which can be prevented via expensive iron chelation therapy. During long-term follow-up, the efficacy of thalidomide in terms of hematological response was stable and sustained over time, and no reduction in response was observed, signifying its aided benefit to TDT patients requiring continued treatment.

The efficacy of thalidomide reported in our study in terms of complete blood transfusion independence in 76.7% patients is consistent with a recently published meta-analysis reporting pooled response rate of nine studies, showing complete cessation of blood transfusion in 52% of TDT patients^3^. Similarly, the significant improvement in mean Hb level (1.4 g/dL at 6th month and 2 g/dL at 30th month) observed in our study is also in line with the findings of a recent study, showing a mean increase of 1.5 g/dL in Hb levels following thalidomide therapy^3^. Another study reported complete cessation of transfusion among 65.9% of patients, and a median increase of 1.6 g/dL Hb level following treatment with thalidomide and HU combination^25^. The observed improvement in Hb level and cessation of blood transfusion may be attributed to HbF inducing properties of thalidomide, thereby improving α:β globin ratio and ineffective erythropoiesis^3^. Additionally, thalidomide regulates erythropoiesis at various stages such as hematopoietic progenitor/stem cells, cell differentiation, increasing the life span of RBC, and globin synthesis^26^ which is evident from our study showing a significant increase in RBCs and its indices (MCV, MCH, and hematocrit). These findings are also consistent with a previous study reporting that increase in Hb level was accompanied by increase in RBCs and indices such as MCV, MCH, hematocrit^27^. The RDW significantly increased at 3 months till 30 months of treatment which is consistent with the findings of khan et al., reporting significant increase in RDW^28^. No significant difference in WBC levels was observed during the study period, which is consistent with previous reports^25,26^. Furthermore, in our study, in order to assess the sustained Hb response of thalidomide, following de-escalation of the dose in a number of cases among excellent responders, and as anticipated, Hb levels dropped significantly in the following two to three months' time. Thus, providing further confirmation of thalidomide role in elevating Hb levels among TDT patients.

A significant reduction in mean serum ferritin level and LDH level was observed during the study period. The observed reduction in serum ferritin level could be attributed to reduced transfusion requirements as well as improved erythropoiesis and increased RBC production. While, a significant decline in LDH level may be attributed to a decrease in hemolysis due to the increased life span of RBCs by thalidomide^26^. In the literature, eight studies reported pre-post serum ferritin levels, of which seven studies reported a significant decrease in mean serum ferritin levels^13–17,25,29^, only one study reported a significant increase in mean serum ferritin levels after treatment with thalidomide among TDT patients^18^. Our study demonstrated a highly significant reduction in spleen and liver size during the study period as reflected by a significant decline in serum ferritin levels and blood transfusions. Morever, our study also demonstrated significant (p ≤ 0.001) increase in mean weight of patients following treatment with thalidomide at 6 months (16.4 kg) and 30 months (21.1 kg) of treatment.

A significant decline in total bilirubin levels following treatment with thalidomide was observed at 6th month of treatment, whereas, no statistically significant difference was found at 30th month of treatment. These findings are in contrast with the findings of Ansari et al., who reported no statistically significant difference following treatment with thalidomide and HU for 6 months^25^. A study by Yang et al., also reported no significant difference in total bilirubin levels after treatment with thalidomide for 24 months in TDT patients^18^. These inconsistencies may be due to less number of patients in the mentioned studies. Our study also demonstrated a significant decline in ALT levels at 6th month till 18 months of treatment. These findings are in contrast with the findings of a study, showing no significant difference in ALT levels after 6 months of treatment with thalidomide and HU combination, and instead reported an increase in ALT levels^25^. Another study by Yang et al., also reported no significant changes in ALT levels after 24 months of treatment with thalidomide^18^. Significant reduction in ALT levels till 21 months of treatment may be attributed to a highly significant decline in serum ferritin levels because there is a positive correlation between serum ALT and ferritin^27^. As far as kidney function is concerned, our study demonstrated no significant changes in creatinine levels, which is in line with the findings of other studies^18,25^. During the study period, thalidomide had no unfavorable effects on both liver and kidney functions instead significant reductions in the ALT and uric acid levels were observed.

The adverse events of thalidomide were mainly mild and tolerable, demonstrating that thalidomide could be a promising drug for TDT. Mild adverse events were reported in 9% of patients, of which constipation was the most frequent adverse event which resolved after symptomatic treatment. These findings are consistent with previous reports of thalidomide^3,12,14,15,25,26,29^. Six patients developed neutropenia; of which, one patient developed neutropenia at 12th month, and five patients at 30th month of treatment. These findings are consistent with other studies which also reported neutropenia collectively in a total of n = 29 patients, the frequency of which is higher than our study (n = 06)^12,14,25^. This study confirms an impact of thalidomide on neutrophil count which is a recognised side effect of thalidomide. In the current study, less number of neutropenia cases and its occurrence upon long-term use may be attributed to the lower dosage of thalidomide used compared to other studies. Serious adverse events such as thrombosis were reported in four patients, of which two patients experienced portal vein thrombosis, and two cerebral vascular accidents that led to the withdrawal of thalidomide treatment. However, both the PVT and CVA resolved among all the affected patients following thalidomide cessation within 2 weeks’ time. These patients were continued on anti-coagulant therapy for a period of 6 months alongside adequate transfusion. These findings are consistent with other studies which also reported central venous thrombosis in one patient^12^ and deep venous thrombosis in two patients^17^. Upon long-term use of thalidomide only mild and tolerable adverse events were reported, demonstrating that thalidomide is a promising drug for patients with TDT refractory to HU.

In light of the findings of this study including attainment of transfusion independence by a large number of patients (76.7%), a significant decline in serum ferritin and LDH levels, reduction in spleen size and liver size, improvement in Hb level and no unfavorable effects of thalidomide on renal and hepatic function, we consider thalidomide, a promising drug for improving Hb levels and reduction/cessation of transfusion requirement in TDT patients. Furthermore, repositioning of thalidomide to increase HbF in β-thalassemia appears very promising based on the following considerations: first, there is a clear clinical pharmacological target, that is an increase of HbF; second, there is no doubt that an increase of HbF decreases transfusion burden; and lastly, there is robust evidence in the literature that in case of HU, in vitro response predicts in vivo response. To this end, thalidomide is particularly the most relevant drug that can be administered in TDT patients based on the proven long-term safety and efficacy observed in this study.

### Limitations

Limitation of the study includes lack of a control group, single centre study, and inability to assess the effect of thalidomide on HbF levels, TSH levels and iron status in each organ. Moreover, we could not assess the pre-post thalidomide quality of life of patients; however, a considerable number of patients achieved transfusion independence; thereby, their quality of life has certainly improved.

## Conclusion

This study has demonstrated the promising role of thalidomide in achieving transfusion independence with proven long-term safety and efficacy in HU refractory TDT patients. The quick response of patients (~ 2 months), long-term safety, easy availability, and affordability especially in low-and-middle income countries, make it a rational therapeutic modality in TDT patients. Based on the study findings, we conclude that thalidomide is an effective and well-tolerated drug in TDT patients. However, the safety of long-term use of thalidomide in TDT patients is not guaranteed and further longer-term prospective randomized controlled studies are needed to provide safety evidence.

## Data Availability

The datasets used and/or analyzed during the current study are available from the corresponding author on reasonable request.
